# FKBP5, a Modulator of Stress Responses Involved in Malignant Mesothelioma: The Link between Stress and Cancer †

**DOI:** 10.3390/ijms24098183

**Published:** 2023-05-03

**Authors:** Giovanni Cugliari

**Affiliations:** Department of Medical Sciences, University of Turin, 10126 Turin, Italy; giovanni.cugliari@unito.it

Malignant pleural mesothelioma (MPM) is a rare tumour characterized by a long latency period after asbestos exposure and poor survival. Due to the complexity of the risk assessment of exposed subjects, the studies focused on the biological mechanisms need to be improved. Stress-related measures are involved in a multitude of disease phenotypes, including cancer.

An important modulator of stress responses is FK506-binding protein 51 (FKBP5/FKBP51), which, among other functions, acts as a co-chaperone that modulates glucocorticoid receptor (GR) activity. These results suggest that FKBP5 may be a key factor in the stress response and that transcriptomic data can provide insight into stress-related pathophysiology [1].

A recent list of reports indicates a strong association of FKBP5 functions with several neurological diseases, including posttraumatic stress disorder (PTSD) [2,3,4]. Epigenetic activation of the FKBP5 gene has increased stress sensitivity and the risk of psychiatric disorders [5]. By modulating GR signaling, FKBP5 has the potential to modulate the actions of glucocorticoids, hormones with pleiotropic effects that can affect essentially every body tissue [6]. Although in psychiatry and neuroscience, FKBP5 is most discussed as a modulator of glucocorticoid signaling, it is important to highlight that it also interacts with a host of other molecular partners, affecting several cellular processes. However, none of the GWAS meta-analyses showed strong associated signals for this genetic locus yet.

More consistent are reports of FKBP5 × specific environmental stress interactions altering the risk for psychiatric disorders. Furthermore, FKBP5 functions have also been correlated with multiple other diseases and processes, including type 2 diabetes, adipogenesis, fatty acid metabolism, and cancers [7]. In several cancers, a strong negative correlation of FKBP5 expression with a severity of disease has been observed [8,9,10,11]. Epigenetics can represent one concrete possibility to improve the mechanical characterization of the disease with the goal of early detection and prognosis stratification.

DNA methylation differences in white blood cells (WBCs) between MPM cases and asbestos-exposed cancer-free controls highlighted some interesting differences [12], including asbestos exposure-related hypo-methylation of FKBP5 in the top marker of risk assessment; interaction analysis showed that considering DNAm levels at FKBP5 together with asbestos exposure levels may help to better define MPM risk for asbestos-exposed subjects [13]. Another recent paper identified hypomethylation of the same CpG in FKBP5 as a predictor of overall survival in MPM cases with better performance than traditional inflammation-based scores such as lymphocyte-to-monocyte ratio (LMR) [14]. FKBP5 is an immunophilin and has an important role in immunoregulation and protein folding and trafficking. It plays a role in transcriptional complexes and acts as a co-transcription factor, along with other proteins in the FKBP family [15]. During the last few years, the hypothesis of a possible role of FKBP5 in the development and progression of different types of cancer has stemmed from several studies. High protein expression has been linked to either suppression or promotion of tumor growth, depending on tumor type and microenvironment [16,17]. FKBP5 has been involved in the NF-kB and AKT signaling pathways, both of which are implicated in tumorigenesis [18]. Notably, NF-kB appears to be frequently constitutively activated in malignant tumors and involved in the modulation of genes linked to cell motility, neoangiogenesis, proliferation, and programmed cell death [19]. The epigenetic upregulation of FKBP5 could promote NF-kB activation [20]. STAT3-NFkB activity is involved in chemoresistance in MM cells, and NFkB was shown to be constitutively active because of asbestos-induced chronic inflammation [21]. Previous studies conducted on various cancer types showed that upregulation of FKBP5 gene expression is associated with drug resistance [22]. The tissue- and context-specific FKBP5 expression should be considered when examining the consequences of FKBP5 dysregulation and when considering FKBP5 as a candidate therapeutic target. A similar study supported this observation by making FKBP5 an effective biomarker for sensitivity to chemotherapy; patient responses to chemotherapy may be determined by the variation in FKBP5 levels [17]. One study on ovarian cancer cell lines denoted that the upregulation of FKBP5 may increase the resistance to chemotherapeutic agents, whereas the gene silencing sensitized ovarian cancer cells to taxol [23].

Lastly, the risk for stress-related disorders is shaped by complex interactions among multiple environmental stressors and many genes with small individual effects on expressed phenotypes. Elucidating these complex interactions at a systems level is a challenging task but may contribute to improving the holistic understanding of stress-related disorders. Furthermore, aging-related epigenetics measures should show interesting associations between stress-related phenotypes and disease to better characterize clusters of exposed subjects. This editorial further supports the notion that stress can affect cancer outcomes in exposed subjects, perhaps by interfering with neuro mechanisms involved in controlling the oncogenesis pathway for early detection, prognosis, and treatment.
